# Association of aspirin resistance with 4‐hydroxynonenal and its impact on recurrent cerebral infarction in patients with acute cerebral infarction

**DOI:** 10.1002/brb3.1562

**Published:** 2020-02-06

**Authors:** Juan Guo, Jue Wang, Yanxia Guo, Juan Feng

**Affiliations:** ^1^ Department of Neurology Shengjing Hospital China Medical University Shenyang China

**Keywords:** 4‐hydroxynonenal, acute cerebral infarction, aspirin resistance, recurrent cerebral infarction

## Abstract

**Objectives:**

To investigate the association of aspirin resistance (AR) with the plasma 4‐hydroxynonenal (4‐HNE) level and its impact on recurrent cerebral infarction (CI) in patients with acute cerebral infarction (ACI) who were receiving aspirin therapy.

**Methods:**

One hundred and fifty‐four ACI patients who previously received aspirin therapy (100 mg/day) were enrolled. Whole urine (for measuring 11dhTXB2 and creatinine) along with blood (for measuring the plasma 4‐HNE level) were collected at least 7 days after the patients received aspirin. A cutoff of 1500 pg/mg of 11dhTXB2/ creatinine was used to determine AR. A follow‐up period to monitor recurrence CI events was 1 year. In addition, blood testing was performed when the patients were first admitted to hospital.

**Results:**

Forty‐six of the 154 enrolled patients (29.9%) were found to be AR. No statistical difference in age, sex, hypertension, diabetes mellitus, coronary disease, smoking status, NIHSS score, TOAST classification, platelet count, thrombocytocrit, LDL‐C, HDL‐C, TG, and TC was found between the AR and aspirin‐sensitive (AS) patients, but the plasma 4‐HNE level was found to be higher in the AR patients than AS patients (*p* < .05). Multiple logistic regression analysis showed that the 4‐HNE level was associated with a higher risk of AR (OR = 1.034; 95% CI = 1.011–1.058; *p* < .05). Moreover, 1‐year follow‐up showed that AR was more prevalent in patients with recurrent CI (26 (56.6%)) than those without (20/(43.5%)) (*p* < .001).

**Conclusions:**

The plasma 4‐HNE level is strongly associated with AR and thus may be a factor contributing to AR. Patients with AR have a greater risk of recurrence CI.

## Graphical abstract

ACI patients with AR have a higher plasma 4‐HNE level, suggesting that 4‐HNE may be a factor contributing to aspirin resistance. And AR has a higher CI recurrence rate compared with the patients without AR.

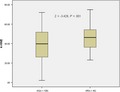

## 1 INTRODUCTION

Aspirin is a widely used antiplatelet agent because low‐dose aspirin can effectively block the platelet cyclooxygenase‐1 (COX‐1) activity and inhibit the synthesis of thromboxane A2 (TXA2) (Chen & Chou, 2018; Lopez et al., 2014; McCullough et al., 2017), which plays an essential anti‐thrombotic role in patients with ischemic stroke. Aspirin resistance refers to the poor responsiveness of some patients to aspirin therapy, which is often described as a failure of aspirin to meet its expected biological effectiveness such as platelet inhibition or an aspirin failure to prevent atherosclerotic thrombus events (Al‐Jabi Samah, 2017; Mason Peter, Jacobs Alice, & Freedman, 2005). It has been reported that between 5% and 45% of the population has aspirin resistance (Mason Peter et al., 2005). Aspirin resistance may increase the risk of recurrence of stroke or myocardial infarction, and is closely related to its severity and infarct size of cerebral infarction (Cheng, Xie, Xu, Chen, & Lian, 2017; Wang et al., 2018; Yi et al., 2016). Clearly, a better understanding of the mechanism of aspirin resistance is critically important to designing new strategies to prevent cerebral infarction, reducing recurrence of cardio‐cerebrovascular diseases.

For patients with acute ischemic stroke, the latest guideline (Powers et al., 2018) recommends the immediate use of aspirin for antiplatelet aggregation benefits. However, aspirin resistance can greatly reduce the effectiveness of aspirin in preventing stroke, meaning that aspirin resistance can significantly increase the risk of recurrent stroke in aspirin‐taking patients. The mechanisms of aspirin resistance are complex and diverse, and most of them are difficult to intervene. However, it has been reported that certain targets produced by oxidative stress play a key role in inducing aspirin resistance, and thus, the therapeutic effect of aspirin could be improved by interfering with these targets (Roger & Johannes, 2006).

4‐hydroxynonenal (4‐HNE) is an unsaturated aldehyde produced mainly by omega‐6 polyunsaturated fatty acids such as linoleic acid and arachidonic acid in lipid peroxidation (Schneider, Tallman, Porter, & Brash, 2001), and it is one of the 5‐aldehyde products in lipid peroxidation (Yang, Sharma, Sharma, Awasthi, & Awasthi, 2003). Benedetti et al. were the first group to report the presence of 4‐HNE in rat liver microsomes during the lipid peroxidation process (Benedetti & Comporti, 1980). Since then, 4‐HNE has been extensively studied (Françoise, 2017). Studies have shown that 4‐HNE is a biomarker of oxidative stress and plays an important role in the pathophysiological process of many diseases (Neven, 2003; Zelzer et al., 2015), including ischemic stroke, neurodegenerative diseases, coronary heart disease, cancer, metabolic syndrome, acute lung injury, and others (Castro, Jung, Grune, & Siems, 2017; Lee et al., 2012; Mason, Charishma, Richard, & Kolliputi, 2016; Xiao, Zhong, Xia, Tao, & Yin, 2017). For example, a study of ischemic stroke patients showed that the concentration of plasma 4‐HNE was higher than that of the control group without ischemic stroke and 4‐HNE could aggravate the ischemic brain damage. It was further suggested that the level of plasma 4‐HNE might be a biomarker of ischemic stroke as it is closely related to oxidative stress and lipid peroxidation (Lee et al., 2012). Guo et al. suggest that oxidative stress is an important factor leading to ischemic damage, and they found that reactive aldehydes like malonaldehyde (MDA) and 4‐HNE produced by oxidized lipids could be detected in all ischemic tissues, including brain tissue, suggesting that the elevated plasma 4‐HNE level may be one of the risk factors of cerebral apoplexy (Guo et al., 2013).

Selley, McGuiness, Jenkin, Bartlett, and Ardlie (1988) were the first to suggest that 4‐HNE could enhance the aggregation of human platelets through various agonists. Subsequently, it was found that in the presence of LDL, the production of 4‐HNE increased during platelet aggregation (Selley, Bartlett, Czeti, & Ardlie, 1998), suggesting that 4‐HNE might cause aspirin resistance due mainly to promoting platelet aggregation. Although the above studies suggested that 4‐HNE might affect platelet aggregation, no study has been done to directly examine the relationship between 4‐HNE and aspirin resistance. So, we hypothesize that 4‐HNE may induce or participate in the occurrence of aspirin resistance as it is one of the key targets of oxidative stress. In this study, we determine the prevalence of aspirin resistance (AR) and its association with the plasma 4‐HNE level and recurrent cerebral in patients with acute cerebral infarction (ACI) who were receiving aspirin. Reporting our findings constitutes the focus of this report.

## 2 METHODS

### 2.1 Patients

Consecutive patients with first ever ACI were recruited for the study, who was admitted to the Neurology Department of our hospital from October 2017 to April 2018. Inclusion criteria were as follows: (a) on aspirin therapy regimens (100 mg daily) for over 1 week (i.e., taking aspirin for at least 7 consecutive days); (b) determination of ischemic infarct by magnetic resonance imaging (MRI); (c) a new focal or global neurological event within 24 hours; (d) the TOAST classification ( Adams et al., 1993) of cerebral infarction included only the atherosclerotic type and the arteriolar type; (e) platelet counts between 100 × 10^9^/L and 500 × 10^9^/L; and (6) informed consents were provided and signed before the performance of the study. The determination of patients’ compliance was achieved by interviewing both patients and their relatives. The patients were excluded from this study for one or more of the following reasons: cardiogenic cerebral infarction; unexplained and other causes of cerebral infarction; medical history of malignant tumor; hematological diseases; chronic inflammatory diseases; autoimmune diseases and other severe dysfunctions of important organs; history of brain trauma and cerebrovascular disease in past 3 months; past platelet function disorders or concurrent administration of additional antiplatelet, anticoagulant, or nonsteroidal anti‐inflammatory medication; recently taking vitamin E, iron ion preparations, or other antioxidation drugs; vitamin E deficiency; and other diseases.

The patient information was collected in the initial stage of this study, covering the following two categories: characteristics of age and sex, and vascular risk factors. Vascular risk factors included smoking habit, hypertension, diabetes mellitus (DM), and coronary heart diseases. Stroke severity of each patient was determined by a neurologist when admitted to hospital, and the National Institutes of Health Stroke Scale (NIHSS) (Williams, Yilmaz, & Lopez‐Yunez, 2000) was used to evaluate strokes severity. Furthermore, the classification of stroke was achieved by using Trial of Org 10172 in Acute Stroke Treatment classification (TOAST).

### 2.2 Blood testing

The fasting venous blood was collected from each patient on the first morning after admission. Platelet count, thrombocytocrit, low‐density lipoprotein cholesterol (LDL‐C), high‐density lipoprotein cholesterol (HDL‐C), triglyceride (TG), and total cholesterol (TC) were measured.

### 2.3 AR and HNE test

Although aspirin resistance can be measured by methods such as light projection aggregation assay, platelet function analyzer, rapid platelet function assay, and others (Stephanie & Rohit, 2008), we used the urine 11‐dehydrothromboxane B2 (11dhTXB2) level to measure AR in this study, as the urine 11dhTXB2 level can be used to measure both aspirin resistance and the inhibitory effect of aspirin on the COX‐1 pathway (Vasudevan et al., 2017). Urine along with fasting blood was collected from each patient with acute stroke on at least seven days after their taking aspirin. The level of 11dhTXB2 in urine was quantified by the enzyme‐linked immunosorbent (ELISA) assay. We used the CORGENIX 11dhTXB2 Test kit (Corgenix, Cat# TXL‐036, RRID:AB_2819213). Urine creatinine was also measured. By following the clinical practice (Lopez et al., 2014), the level of 11dhTXB2 in urine was measured in 11dhTXB2/creatinine. A cutoff of 1,500 was used to determine the status of AR, meaning that a patient was considered as AR with a value ≥1,500 pg/mg, while as aspirin‐sensitive (AS) when the value is smaller than 1,500 pg/mg (Lopez et al., 2014). The blood collected from each patient was converted to plasma, from which the level of human 4‐hydroxynonenal (HNE) was measured by human 4‐hydroxynonenal (HNE) ELISA kit (Cusabio Biotech, Cat# CSB‐E16214h, RRID:AB_2819206).

### 2.4 Follow‐up

During 1‐year period after the first admission, we followed the patients who had been discharged from the hospital using a standard questionnaire, and telephone or household contact by physician investigators every 4 months after admission. The endpoint was stroke recurrence during 1‐year period after stroke onset. Ischemic stroke recurrence was defined as sudden functional deterioration in neurological status with a decrease of NIHSS score of 4 or more, or a new focal neurological deficit of vascular origin lasting >24 hours (Ning, Zhenhua, & Lihong, 2017).

### 2.5 Statistical analysis

Continuous variables were expressed as mean standard deviation while categorical variables were expressed as ratio. To evaluate the differences between mean values of quantitative variables, Student's *t* test was used for parametric variables(age and 4‐HNE).and the Mann–Whitney *U* test for nonparametric variables (NIHSS score, platelet count, thrombocytocrit, LDL‐C, HDL‐C, TG, TC). Categorical variables like two classification variables (sex, smoking habit, hypertension, DM, coronary heart diseases, TOAST) were compared using chi‐square test. In Tables 1, 2, 3, parametric variables in parentheses were showed by mean‐SE and mean + *SE*, nonparametric variables in parentheses were showed by second quartile and fourth quartile, and categorical variables in parentheses were showed by percentage. The ROC curve was drawn to evaluate the ROC analysis of the plasma 4‐HNE content in the evaluation of aspirin resistance, and the plasma 4‐HNE level was used to evaluate the area, sensitivity, specificity of the aspirin resistance curve. Multiple logistic regression model was constructed to identify the associations between AR and 4‐HNE, which allows adjustment for confounding factors (age, sex, and vascular risk factors). Results of regression analyses were expressed as adjusted odds ratios (OR) with 95% confidence interval (CI). SPSS 22.0 (SPSS Inc.) was used for all statistical analyses, *p* < .05 indicates statistical significance. Spearman was used to identify the correlation between 11dhTXB2 and 4‐HNE, and scatter plot suggests a positive correlation between 4‐HNE and 11dhTXB2.

**Table 1:** Comparison of basic data between AR group and AS group

Baseline data	AR	AS	*p*
*N *(%)	46 (29.9)	108 (70.1)	
11dhTXB2/creatinine (pg/mg)	2,101.82 (1,595.99, 2,979.16)	811.80 (576.43, 114.53)	
11dhTXB2 (pg/ml)	988.0 (588.8, 1,637.0)	656.5 (376.0, 1,259.5)	
Male (％)	30 (65.2)	65 (60.2)	.557
Age (year)	64 (53, 76)	62 (51, 73)	.209
Hypertension (％)	36 (78.3)	71 (65.7)	.123
DM (％)	14 (30.4)	39 (27.8)	.738
Coronary disease (％)	5 (10.9)	12 (11.1)	.965
Smoking (％)	13 (28.3)	32 (29.6)	.864
NIHSS score	2 (1, 4.25)	2 (0, 3)	.093
Atherosclerotic CI	20 (43.5)	34 (31.5)	.153
Platelet count	203.5 (172, 243.25)	202.5 (163.75, 235.25)	.530
Thrombocytocrit	0.18 (0.15, 0.2)	0.17 (0.15, 0.21)	.629
LDL‐C	2.615 (2.0675, 3.315)	2.705 (2.11, 3.2)	.709
HDL‐C	1.045 (0.775, 1.1525)	1.01 (0.85, 1.2275)	.510
TG	1.25 (0.865, 2.245)	1.315 (0.9, 1.915)	.746
TC	4.265 (3.38, 4.9775)	4.27 (3.565, 4.8875)	.914

Abbreviations: DM, diabetes mellitus; HDL‐C, high‐density lipoprotein cholesterol; LDL‐C, low‐density lipoprotein cholesterol; NIHSS, National Institutes of Health Stroke Scale; TC, total cholesterol; TG, triglycerides.

**Table 2:** Independent predictors for AR in patients with acute cerebral infarction

	OR	95% CI	*p*
4‐HNE	1.041	1.015–1.067	**.002**
Sex (male)	0.552	0.241–1.266	.161
Age	1.040	1.003–1.079	**.034**
Hypertension	1.742	0.743–4.08	.202
DM	0.810	0.348–1.887	.626
Coronary diseases	0.905	0.268–3.055	.872

Note

*p* values set in boldface indicate statistical significance.

Abbreviation: 4‐HNE, 4‐hydroxynonenal; AR, aspirin resistance; DM, diabetes mellitus.

**Table 3:** Comparison of AR rate between recurrence group and nonrecurrence group

	Recurrence	Non‐Recurrence	*p* value
*N* (%)	39 (25.32)	115 (74.68)	
AR (%)	24 (58.5)	22 (19.5)	<.001
4‐HNE (NG/ML)	40.62 (22.67, 58.57)	41.35 (24.62, 58.08)	.805

Abbreviation: 4‐HNE, 4‐hydroxynonenal; AR, aspirin resistance.

## 3 RESULTS

### 3.1 Characteristics of patients

In this study, a total of 154 patients were examined for ACI. In those patients, the average age was 63 years (53–74 years), and 99 (64.3%) of them were men. The score of 11dhTXB2 ranged from 2.19 to 74.91 pg/ml with an average value of 41.11 pg/ml. Of the 154 enrolled patients, 46 patients were determined to have AR (29.9%), which was determined by the level of 11dhTXB2/creatinine in urine. Table 1 summarizes the characteristics of the 154 patients with AR or AS. Statistical analysis showed that for the patients in the AR and AS groups, there is no statistical difference in their age, sex, hypertension, diabetes mellitus, coronary disease, smoking status, NIHSS score, TOAST classification, platelet count, thrombocytocrit, LDL‐C, HDL‐C, TG, and TC.

### 3.2 AR and 4‐HNE

**Figure 1:**
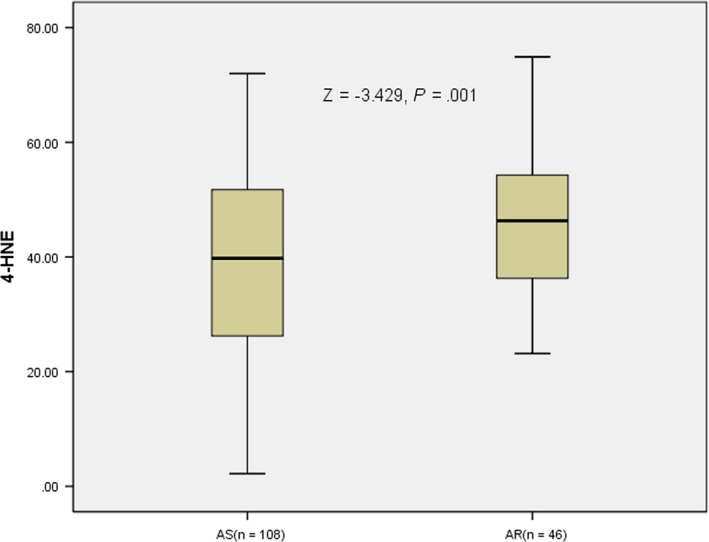
Comparison of Plasma 4‐HNE level between AR group and AS group

**Figure 2:**
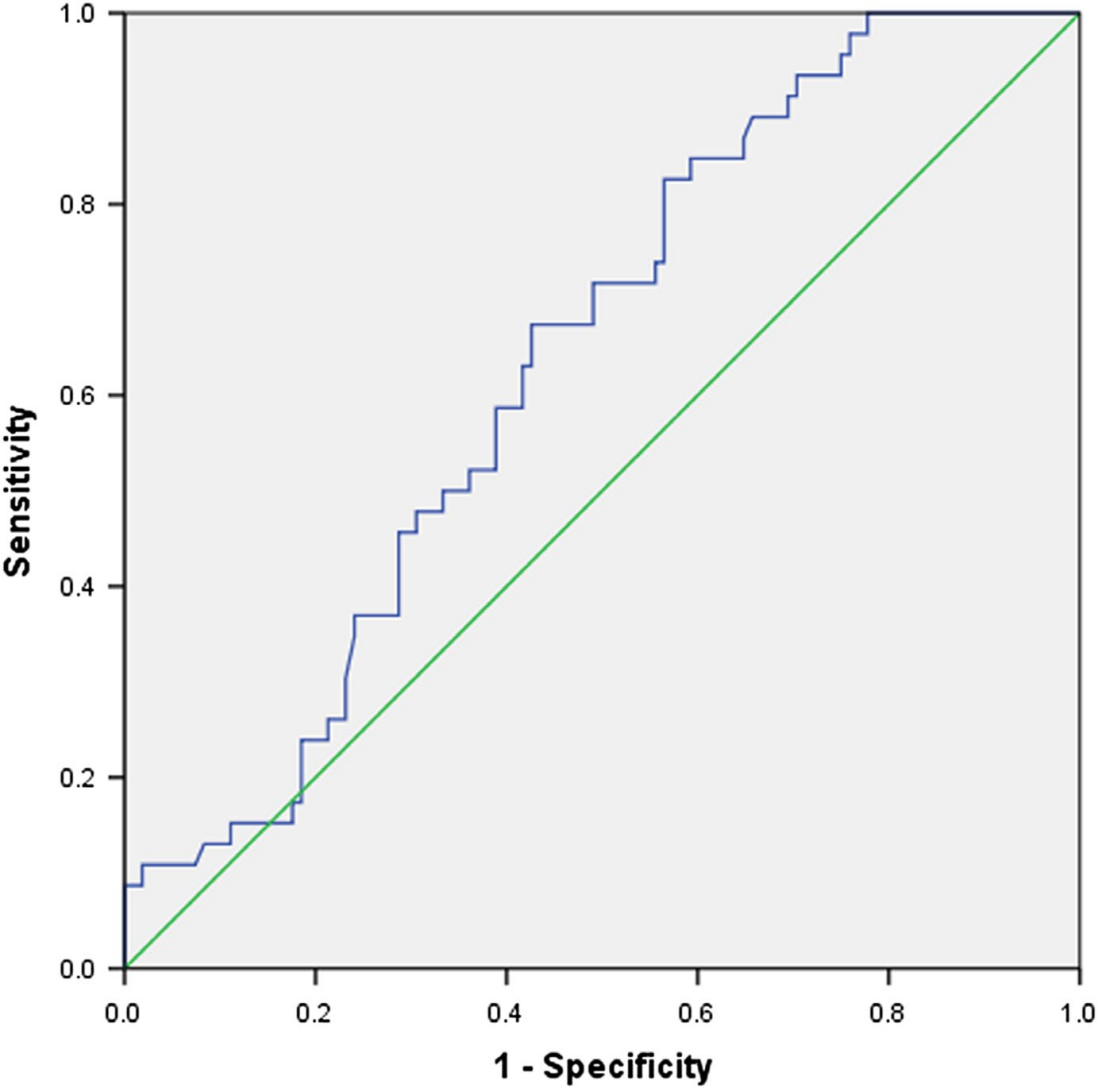
Receiver operator characteristic curve demonstrating sensitivity as a function of 1‐specificity for predicting the AR based on the logistic model incorporating markers (4‐HNE). This logistic model had an area under the receiver operator characteristic curve of 0.637 (95%CI, 0.584–0.725, *p* = .007). The sensitivity of the model was 82.6%, and the specificity of the model was 43.5%, with the optimal cut‐off value of 26.3%

**Figure 3:**
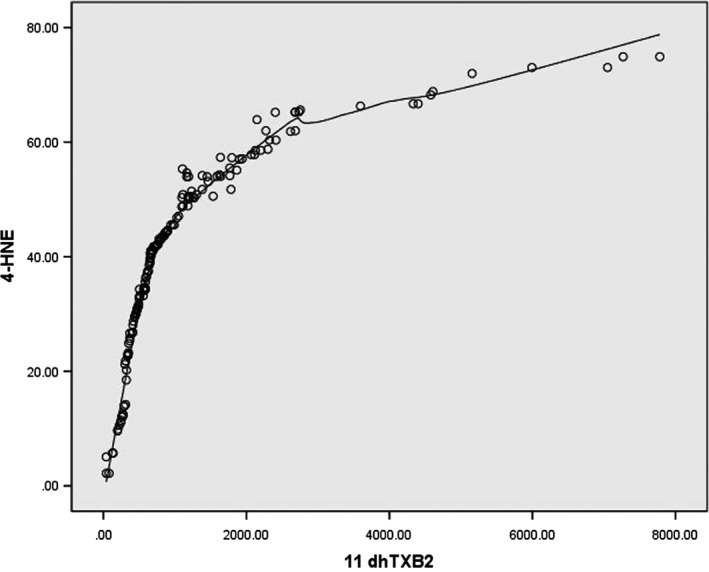
Scatter plot suggests a positive correlation between 4‐HNE and 11dhTXB2

As shown in Figure 1, the median 4‐HNE level was 41.11 ng/ml (24.03–58.19 ng/ml) in the 154 patients with ACI. Importantly, the plasma 4‐HNE level was higher in patients with AR (47.41 [33.95–60.87]) than those without (38.43 [20.64–56.22]), and this difference is statistically significant (*p* < .05). The ROC curve analysis (Figure 2) shows that 4‐HNE is associated with aspirin resistance in patients with acute ischemic stroke (0.637 [95%CI, 0.584–0.725]) (*p* < .05; Figure 2), indicating that 4‐HNE could be used to evaluate the occurrence of aspirin resistance. Multivariate analyses (Table 2) showed that after adjusting for other confounding factors, 4‐HNE may be an independent risk factor for predicting aspirin resistance in patients with acute ischemic infarction (OR = 1.041, [95%CI 1.015–1.067], *p* = .002). This indicates that the higher the plasma 4‐HNE level, the higher the incidence of aspirin resistance after adjusting for other risk factors. Spearman's rank correlation test (Table 4, *r*_s_ = .996) and scatter plot (Figure 3) correlation test suggest a positive correlation between 4‐HNE and 11dhTXB2. Our analysis also showed that an increase in the incidence of aspirin resistance was associated with age (OR = 1.040, [95%CI 1.003–1.079], *p* = .034), but no association was found between the parameters such as sex, hypertension, diabetes mellitus, and coronary disease were not associated and the incidence of aspirin resistance.

**Table 4:** Spearman's rank correlation test

Correlations			4‐HNE	11dhTXB2
Spearman ‘s rho	4‐HNE	Correlation coefficient	1.000	.996^a^
		Sig (2‐tailed)	–	0.000
		*N*	154	154
	11dhTXB2	Correlation coefficient	.996^a^	1.000
		Sig (2‐tailed)	0.000	–
		*N*	154	154

Abbreviation: 4‐HNE, 4‐hydroxynonenal.

a Correlation is significant at the .01 level (2‐tailed).

### 3.3 AR and recurrent CI

Table 3 summarizes the CI rate during the one‐year follow‐up period. All patients were divided into recurrent CI group (50 [32.47%]) and nonrecurrent CI (104 [67.53%]) group according to the follow‐up results. The plasma 4‐HNE level in the recurrent and nonrecurrent groups was compared, but no statistically significant difference was found. However, when we compared the incidence of aspirin resistance in these two groups, it was seen that patients with recurrent CI are more likely to develop aspirin resistance than those with nonrecurrent CI (26 [56.6%] vs. 20 [43.5%], *p* = .000), further providing the evidence that the patients with aspirin resistance are more likely to suffer from recurrent CI.

## 4 DISCUSSION

In this study, we further provide evidence that the ACI patients with AR have a higher CI recurrence rate compared with the patients without AR. Importantly, although it has been suggested that 4‐HNE might affect platelet aggregation (Selley et al., 1998, 1988), no study has been done to directly examine the relationship between 4‐HNE and aspirin resistance in the ACI patients. Our study was the first one to examine such relationship and clearly showed that there is a strong association of 4‐HNE with aspirin resistance in the ACI patients, suggesting that 4‐HNE is associated with AR or may even play an important role in aspirin resistance.

4‐HNE is associated with AR, but an essential question is whether it is a contributing factor affecting platelet aggregation? If the answer is yes, the question is how does 4‐HNE interfere with the efficacy of aspirin by promoting platelet aggregation? Clearly, an understanding of the role that 4‐HNE may play in AR is critically important. Next, we discuss the relationship of 4‐HNE and AR to shed some insights into the potential role 4‐HNE in AR.

It was reported that 4‐HNE is one of the molecules that could induce COX‐2 expression, and as a lipid peroxidation product, it may play a unique role in activating the signal transduction pathway, enhancing the expression of COX‐2 (Koji, (2017)). Another study suggests that 4‐HNE up‐regulates the COX‐2 expression by stabilizing its mRNA through the p38 mitogen‐activated protein kinase signaling pathway (Koji & Takeshi, 2003). Under normal physiological conditions, COX‐2 usually does not exist or its level is very low, but through the stimulation by the factors like pro‐inflammatory cytokines, carcinogens, and growth factors, COX‐2 can be expressed in vascular endothelial cells, smooth muscle cells, and platelets (Gong et al., 2017; Paola, 2003). The production of COX‐2 can lead to an increase in the aspirin‐insensitive TXA2 synthesis (Fontana, Zufferey, Daali, & Reny, 2014), suggesting that an increase in expression of COX‐2 induced by 4‐HNE may reduce the efficacy of aspirin in stroke patients by inducing vasoconstriction and platelet aggregation through the synthesis of aspirin‐insensitive TXA2. It is noted that although aspirin reduces the production of TXA2 by inhibiting COX, it has different inhibitory effects on COX‐1 and COX‐2. Aspirin inhibits COX‐1 effectively, but has weak effect on COX‐2 (McCullough et al., 2016).

Furthermore, it was reported that the resistance of young men to aspirin is related to endothelial dysfunction, which may be due to oxidative stress induced by lipid peroxidation, increasing the aspirin‐insensitive TXA2 biosynthesis (Doroszko et al., 2015). It was also reported that oxidative stress promotes platelet aggregation through the production of a lipid peroxidation biomarker of 8‐isoprostaglandin F2α, which affects the therapeutic effect of aspirin by oxidizing it (Ayala, Muñoz, & Argüelles, 2014). Like 8‐isoprostaglandin F2α, 4‐HNE is also a biomarker of lipid peroxidation. Considering the role of 8‐isoprostaglandin F2α in aspirin resistance by oxidizing it (oxidation stress), we speculate that 4‐HNE could play a similar role in oxidation of aspirin, leading to aspirin resistance.

Taking the above evidences together, we speculate that 4‐HNE may be a contributing factor leading to aspirin resistance. However, it must be noted that although 4‐HNE may have effects on aspirin resistance, leading to platelet aggregation, the exact mechanism is not clear, and thus, more studies are warranted to elucidate the role of 4‐HNE in aspirin resistance.

This study further provides evidence for the association of aspirin resistance with recurrence of cerebral infarction. In clinical practice, it is aware that for patients with AR, the recurrence CI is more common compared with those without AR after patients take aspirin. However, because the mechanism of aspirin resistance is not clear, it is impossible to achieve intervention to improve the efficacy of aspirin. Therefore, it is important to explore the mechanism of aspirin resistance. 4‐HNE, as a biomarker of oxidative stress, exists in plasma and is relatively easy to detect. It can be used to detect the level of aspirin by simple manipulation, which may be used to reflect the extent of aspirin resistance. We have reported that 8‐isoprostaglandin F2α induced by oxidative stress plays an important role in aspirin resistance. Our study shows that the plasma 4‐HNE level, a biomarker of oxidative stress, is also related to aspirin resistance. Therefore, the effect of oxidative stress on aspirin resistance should be further confirmed. Antioxidation‐related drugs may be used to interfere with aspirin resistance. We believe it is possible to interfere with its expression by means of antioxidation‐related drugs in future, so as to provide a means for clinical evaluation, prevention, and treatment of aspirin resistance.

There are some limitations in our study. Firstly, the number of cases included in this study is not very large. Therefore, the recurrence rate of cerebral infarction by follow‐up is higher than that of other large studies (Huang et al., 2008; Kim et al., 2018) in China, which include hundreds of patients. Secondly, the plasma 4‐HNE level was collected only once, which cannot reveal its association with AR over an entire period of one‐year follow‐up. Thirdly, since 11dhTXB2 was also only measured once, we did not know whether the patient was AR or AS at the time of recurrence.

## 5 CONCLUSIONS

In conclusion, our study shows that ACI patients with AR have a higher plasma 4‐HNE level, suggesting that 4‐HNE may be a factor contributing to aspirin resistance. Our study also shows that the patients with AR have a higher CI recurrence rate compared with the patients without AR.

## CONFLICT OF INTEREST

None declared.

## Data Availability

The data that support the findings of this study are available on request from the corresponding author. The data are not publicly available due to privacy or ethical restrictions.
